# Oncocytic Adrenocortical Carcinoma with Somatic Pathogenic Variants of *NF1* and *TP53* Genes in a Young Adult Harboring a Germline Likely Pathogenic Variant in *CEL* Gene: From Hyperandrogenemia of Dual (Adrenal–Ovarian) Cause to Oocyte Preservation and Mitotane Initiation

**DOI:** 10.3390/diagnostics16121935

**Published:** 2026-06-22

**Authors:** Mara Carsote, Augustin Dima, Oana-Claudia Sima, Ana-Maria Gheorghe, Mihai Costachescu, Elena-Emanuela Braha, Sorina Violeta Schipor, Dana Manda, Andrei Muresan, Anda Dumitrascu, Adrian Ciuche, Laura Dracea, Teodor Ionut Constantin, Dana Terzea

**Affiliations:** 1 Department of Endocrinology, “Carol Davila” University of Medicine and Pharmacy, 020021 Bucharest, Romania; carsote_m@hotmail.com; 2 Department of Clinical Endocrinology V, “C.I. Parhon” National Institute of Endocrinology, 011863 Bucharest, Romania; oana-claudia.sima@drd.umfcd.ro (O.-C.S.); ana-maria.gheorghe@drd.umfcd.ro (A.-M.G.); 3 PhD Doctoral School, “Carol Davila” University of Medicine and Pharmacy, 020021 Bucharest, Romania; 4 Department of Surgery, “Dr. Carol Davila” Central Military University Emergency Hospital, 010825 Bucharest, Romania; dimagusti@gmail.com; 5 Department of Radiology and Medical Imaging, “Dr. Carol Davila” Central Military University Emergency Hospital, 010825 Bucharest, Romania; 6 Research Department, “C.I. Parhon” National Institute of Endocrinology, 011863 Bucharest, Romania; elena.braha@parhon.ro (E.-E.B.); dana.manda@parhon.ro (D.M.); andrei.muresan@parhon.ro (A.M.); 7 Department of Radiology and Medical Imaging, “C.I. Parhon” National Institute of Endocrinology, 011863 Bucharest, Romania; anda.dumitrascu@gmail.com; 8 Department 4—Cardio-Thoracic Pathology, Thoracic Surgery II Discipline, “Carol Davila” University of Medicine and Pharmacy, 020021 Bucharest, Romania; adrian.ciuche@umfcd.ro; 9 Thoracic Surgery Department, “Dr. Carol Davila” Central Military University Emergency Hospital, 010825 Bucharest, Romania; 10 Gynera Fertility Center, 020308 Bucharest, Romania; laura.dracea@gynera.ro; 11 Oncology Outpatient Compartment, “C.I. Parhon” National Institute of Endocrinology, 011863 Bucharest, Romania; teodorc.med@gmail.com; 12 Department of Pathology, “C.I. Parhon” National Institute of Endocrinology, 011863 Bucharest, Romania; danaterzea@gmail.com; 13 Oncoteam Diagnostics, 012244 Bucharest, Romania

**Keywords:** adrenalectomy, mitotane, ovary, androgens, Ki67, reticuline, *CEL* gene, *NF-1* gene, *TP53* gene, polycystic ovary syndrome, Helsinki score, NGS, Lin–Weiss–Bisceglia score, HOMA-IR, glycemia, insulin, testosterone, DHEA-S, CT, MRI, MMR, MSI

## Abstract

The oncocytic variant of adrenocortical carcinoma (OACC) represents an exceptional type of adrenal malignancy, with heterogenous presentation. Currently, the genetic and molecular spectrum remains an open matter. A 20-year-old adult was accidentally found with a 7.2 cm adrenal tumor and underwent an open right adrenalectomy with OACC confirmation. Post-adrenalectomy positron emission tomography/computed tomography was negative. Immunohistochemistry was positive for calretin, inhibin, steroidogenic factor 1; Ki67 of 20%. Microsatellite instability was 7.61. Lin–Weiss–Bisceglia score showed 2 major criteria [mitoses 6/50 HPF + positive atypical mitoses], the reticuline algorithm (disrupted reticuline network + mitoses 6/50 HPF) was consistent for a malignant behavior, the Helsinki score was of 48. Next generation sequencing identified a likely pathogenic variant of *CEL* gene (heterozygote, c.539-2A>G) in peripheral blood and two pathogenic variants in the tumor: exon 48, *NF1* gene [c.7159_7164del p.(N2387_F2388del)] and exon 6, *TP53* gene [c.596delG p.(G199Efs*48)]. Polycystic ovary syndrome type A has been diagnosed as teenager with no phenotype change before the tumor detection. After surgery, oocyte retrieval and cryopreservation upon ovarian stimulation protocol (OSP) was performed before starting mitotane therapy. To the best of our knowledge, this is a novel genetic configuration in OACC with an impact on prognosis to be determined. Hyperandrogenemia stands on a dual source (potential *CEL*-driven insulin resistance for the ovary and OACC-originating for the adrenal glands). Also, this is the first case to receive OSP in OACC, noting that a tailored multidisciplinary management is mandatory.

**Figure 1:**
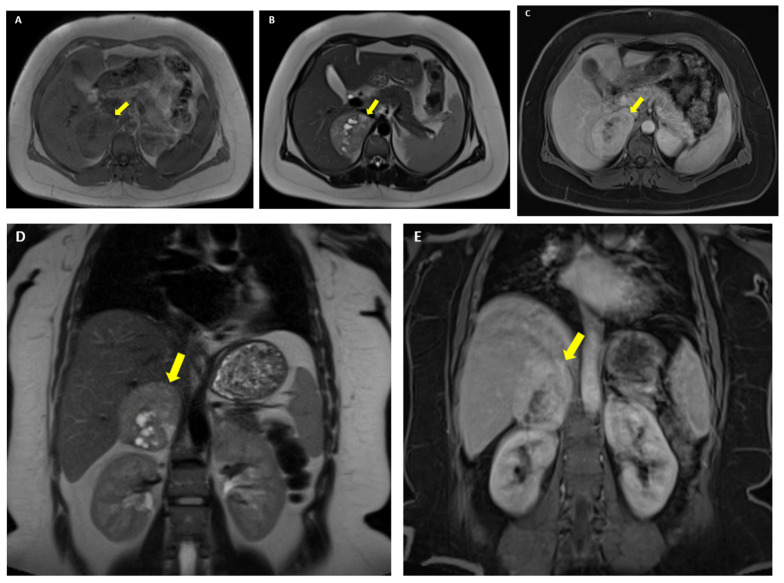
This is a 20-year-old adult who presented an episode of non-specific abdominal pain, initially regarded as a non-lithiasic cholecystic crisis. After abdominal ultrasound, further imaging evaluation was performed under these circumstances and led to the identification of a large right adrenal tumor at magnetic resonance imaging (MRI) [(**A**–**E**). Right adrenal tumor (yellow arrow) (**A**). MRI scan native phase, T1 WI FSE, axial plane: tumor of 7.2 by 5.3 by 6.9 cm, well-shaped, hypointense, without signal drop out on opposed phase sequence, with multiple cystic areas of maximum diameter 1.7 cm (the tumor compresses the medial margin of the right hepatic lobe and the inferior vena cava, while being well-separated from these structures); (**B**). MRI scan native phase, T2 WI HASTE, axial plane: tumor with intermediate/hyperintense signal, without signal drop out on opposed phase sequence; (**C**). Contrast enhanced MRI scan, T1 WI VIBE Dixon water-only, axial plane: tumor with hypointense signal, without signal drop out on opposed phase sequence, with moderate and slowly progressing gadolinium enhancement; multiple cysts insight the solid mass; (**D**). MRI scan native phase, T2 WI HASTE, coronal plane; (**E**). contrast enhanced MRI scan, T1 WI VIBE Dixon water-only, coronal plane].

**Figure 2:**
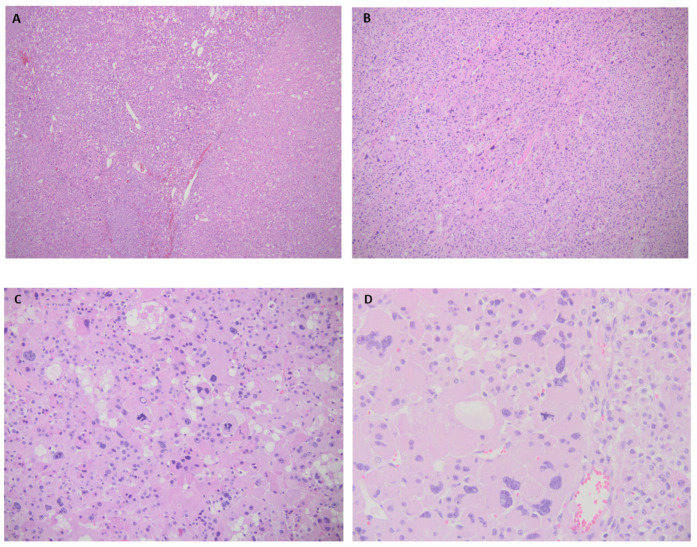
Noting the initial presentation with unusual abdominal pain (without a clear cause) and the potential connection with the newly detected large adrenal mass, the patient soon underwent an open right adrenalectomy. Pre-operatory, she presented normal blood pressure and a pheochromocytoma was ruled out based on normal plasma metanephrines of 0.09 (Normal < 0.36) nmol/L and normetanephrines of 0.65 (Normal < 0.71) nmol/L. The histological report confirmed a well-shaped, encapsulated adrenal tumor with dense cellular proliferation and focal cystic areas (sized of 8.2 by 7.8 by 4 cm; weight of 184 g). The cells were round, oval or polygonal shape, of medium or large size (multiple nests and islands of cells); with rich eosinophil cytoplasm, focal vacuolization, round or oval nuclei, nuclei with bizarre forms, nuclei with intra-nuclear inclusions, and relatively frequent mitoses [6/50 high power field (HPF)], atypical mitoses, reduced stroma, frequent vessels and areas of hemorrhage. No capsular, vascular, peri-adrenal (fibro-adipose tissue) invasion, nor necrosis were detected. These findings were consistent with the diagnosis of an adrenocortical carcinoma of oncocytic variant [(**A**–**D**). Histological analysis (hematoxylin-eosin): (**A**). magnification 4×; (**B**). 10×; (**C** and Figure S1). 20×; (**D** and Figure S2). 40×].

**Figure 3:**
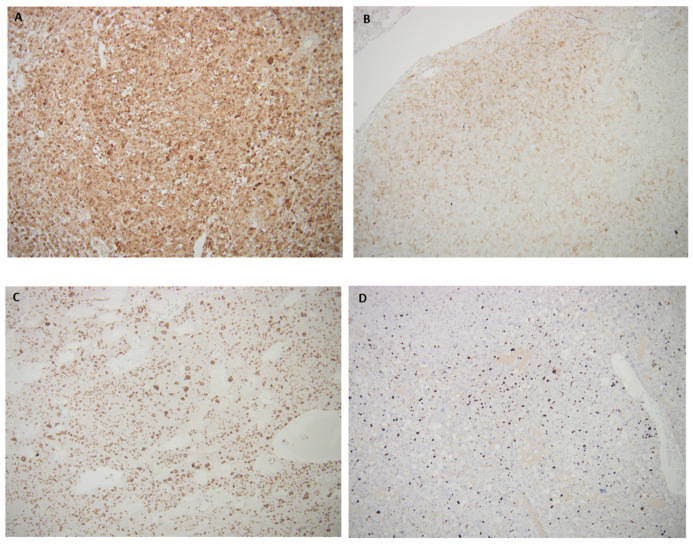
Immunohistochemistry analysis showed a positive staining in tumor cells for calretin, inhibin, steroidogenic factor 1 (SF1); negative chromogranin A; positive p53 of 5% in wild-type tumor cells; positive PHH3 (phospho-histone H3) in 6% of the tumor cells; and a Ki67 proliferation index of 20%. [(**A**). Positive calretin in tumor cells, magnification 10×; (**B**). Positive inhibin in tumor cells, magnification 10×; (**C** and Figure S3). Positive SF1 in tumor cells, magnification 10×; (**D** and Figure S4). Ki67 of 20%; magnification 10×]. MMR (Mismatch Repair Proteins) protein testing (MLH1, MSH2, MSH6, PMS2) showed a normal nuclear expression, as well as microsatellite stability (MSS). There was an overall normal MMR immuno-profile according to a microsatellite instability (MSI) score of 7.61 (significance: MSS stands for a score < 24, while high MSI is reflected by a score > 25).

**Figure 4:**
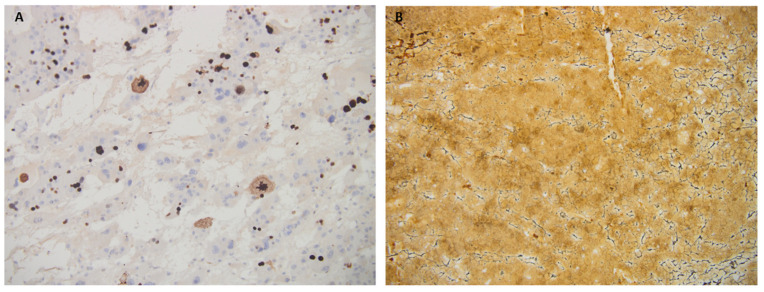
Lin-Weiss-Bisceglia score included two major criteria (mitoses 6/50 HPF + positive atypical mitoses). The reticuline algorithm showed a disrupted reticuline network. The Helsinki score was 48 based on 3 points × 6 (for mitoses 6/50 HPF) plus 20 (the value of Ki67) with ENSAT stage II [(**A**). Immunohistochemistry stain: Ki67 of 20%, magnification 20×; (**B**). Disrupted reticuline network, magnification 10×].

**Figure 5:**
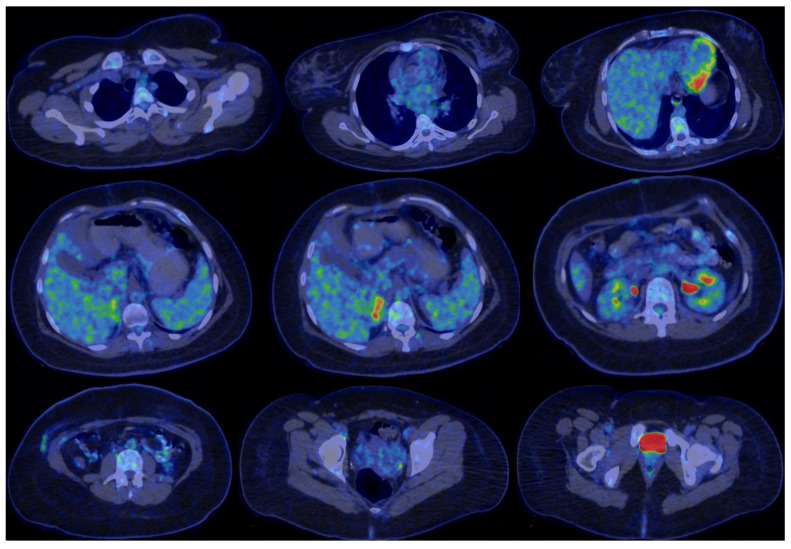
After post-adrenalectomy identification of the adrenal cancer, the patient underwent positron emission tomography/computed tomography (PET/CT) and found negative for an active disease. PET/CT scan: no pathological metabolically active foci in the left adrenal gland right adrenal region thoracic, abdominal or pelvic level. Figure S5. Integrative Genomics Viewer (IGV version 2.16.0) for the CEL gene; Figure S6 c.539-2A>G. Next generation sequencing (NGS-extended tumor germline panel for 4813 genes) was performed from the peripheral blood (Illuma MiSeq, kit TRusight One Sequencing Panel) and identified a likely pathogenic variant in *CEL* gene (splice acceptor, heterozygote, c.539-2A>G, chromosome 9) according to The American College of Medical Genetics and Genomics (ACMG). In addition, NGS in the tumor (Oncomine Comprehensive Assay Plus) established a somatic characterization and identified a pathogenic variant in exon 48 of *NF1* gene [c.7159_7164del p.(N2387_F2388del)] and a pathogenic variant in exon 6 of *TP53* gene [c.596delG p.(G199Efs*48)]. The germline variant identified in *CEL* gene (c.539-2A>G, NM_001807.6) is a novel heterozygous splice site acceptor variant, not reported in the literature or in public databases (gnomAD, UCSC, 1000 Genomes, ExAC). The coverage for this specific locus was high (148X) and the allele balance highly confident (variant allele frequency of 48.65%). This variant is classified as likely pathogenic fulfilling PVS1 and PM2 ACMG criteria. In silico predictions for this variant show an aggregated score prediction as deleterious (SpliceAI score of 0.99 indicates splice-altering/strong prediction; Mutation Taster classifies this variant as deleterious). The germline variant identified by NGS was visually inspected in Integrative Genomics Viewer (IGV) and met established technical quality metrics, including adequate coverage and sequencing quality parameters, therefore not requiring additional confirmatory testing.

**Figure 6:**
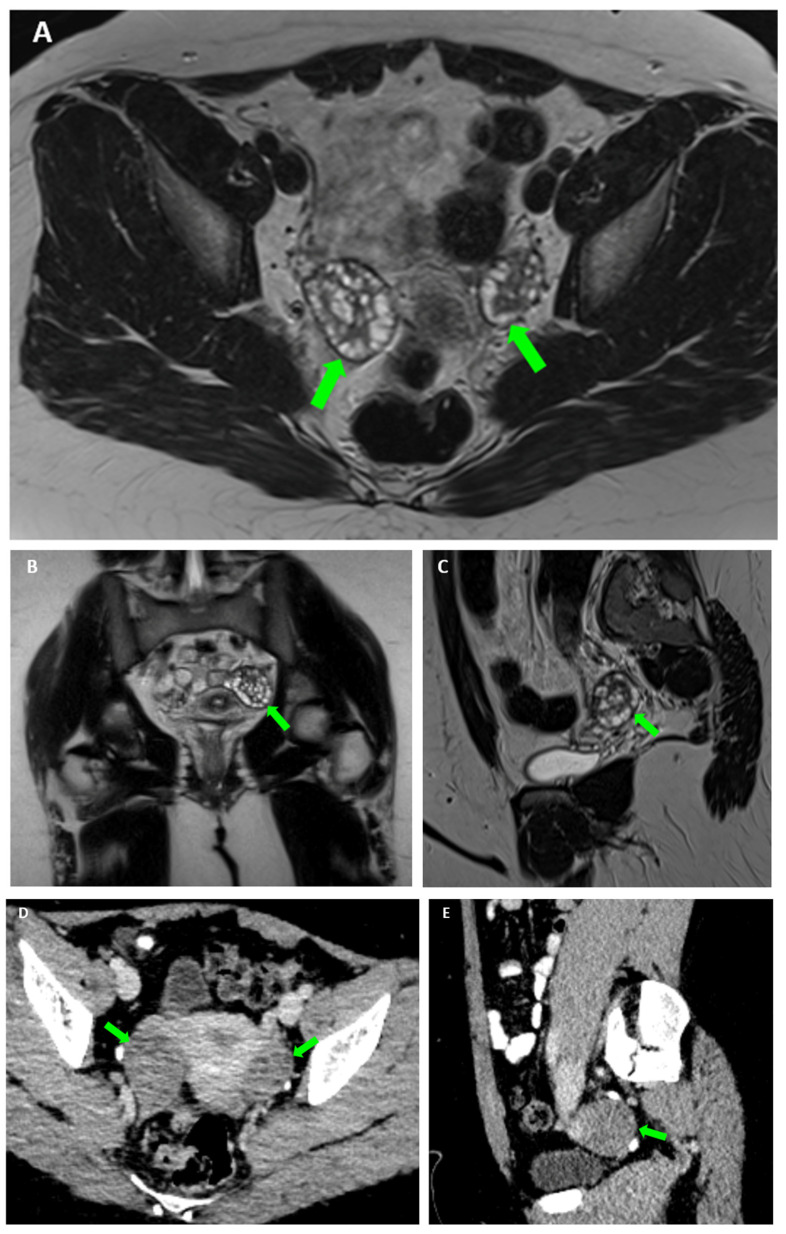
The medical history included spontaneous menarche since the age of 13, irregular menses (3 to 5 cycles per year), in association with obesity and acne. She was diagnosed with polycystic ovary syndrome type A and follow-up during teenager years as outpatient, undergoing a normal puberty with an adult height according to the mid-parental height. Over the years, no particular long-term medication was administered and the weight excess was apparently resistant to usual hypocaloric diets. Within the months before the tumor detection, no distinct clinical clue was retrospectively identified, neither a change in the phenotype. At the timing of adrenalectomy, the body mass index was 37 kg/sqm and no overt Cushing’s syndrome was pointed out. The medical records with respect to the biochemical and hormonal assays revealed at the age of 19 (a few months before the tumor removal): insulin resistance based on a Homeostatic Model Assessment of Insulin Resistance (HOMA-IR) of 4 (Normal < 2) without diabetes/prediabetes; increased dehydroepiandrosterone sulphate (DHEA-S) of 604 (Normal: 148–407) µg/dL and elevated total testosterone level of 5.42 (Normal < 1.67) nmol/L, with normal thyroid function and prolactin [(**A**–**C**). Pre-adrenalectomy aspect of the ovaries (green arrows) at MRI: right ovary of 3.2 by 3.9 by 3.3 cm and left ovary of 2.7 by 3.6 by 4.1 cm (both with inhomogeneous structure and multiple peripherally distributed microcysts); (**A**). native phase, T2 WI FSE, axial plane; (**B**). native phase, T2 WI HASTE, coronal plane (left ovary); (**C**). native phase, T2 WI FSE, sagittal plane (left ovary). (**D**,**E**). Post-adrenalectomy aspect of the ovaries (green arrow) at CT scan: right ovary of 3.3 by 4.0 by 3.8 cm and left ovary of 2.7 by 5.1 by 4.7 cm, both with a micro-polycystic structure; (**D**). delayed phase, axial plane; (**E**). delayed phase, sagittal plane]. After unilateral adrenalectomy, the blood pressure remained normal and the patient did not experience any adrenal insufficiency. Hormonal panel showed a normal adrenocorticotropic hormone (ACTH) of 16.03 (Normal: 7.2–63.3) pg/mL, plasma morning cortisol of 11.5 (Normal: 6.2–19.4) µg/dL, DHEA-S of 90.1 (Normal: 148–407) µg/dL, total testosterone of 0.66 (Normal < 1.67) nmol/L, follicle-stimulant hormone (FSH) of 2.25 mUI/mL, luteinizing hormone (LH) of 6.64 mUI/mL, and estradiol of 40.83 pg/mL (in amenorrhea). A 75g oral glucose tolerance test (OGTT) ruled out an impaired glucose profile based on fasting glycemia of 86 mg/dL, at one hour of 136 mg/dL, and at two hours of 107 mg/dL, while glycated hemoglobin A1c remained normal of 5.2 (Normal: 4.8–5.9) %, as found pre-operatory. Fasting insulin (of 13.2 µUI/mL) increased at 126.3 µUI/mL, respectively, at 42.28 µUI/mL at one hour, respectively, at two hours during OGTT. HOMA-IR improved at 2.8, but remained higher than normal (<2).

**Figure 7:**
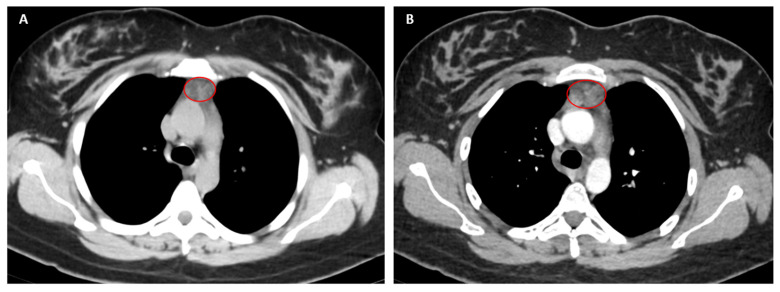
CT scan was performed two months after adrenalectomy and showed no right adrenal remnants/recurrence, neither any lymph node involvement. Accidentally, a nodule of 3.3 by 1.8 cm was identified within the fat tissue component in the thymus area indicating a thymic remnant. This finding was not suspected for a thymus tumor/malignancy; thus, no surgical approach was recommended, only serial CT scan surveillance. [(**A**,**B**). CT scan: thymus remnant (red cercle). (**A**). native phase, axial plane; (**B**). arterial phase, axial plane].

**Figure 8:**
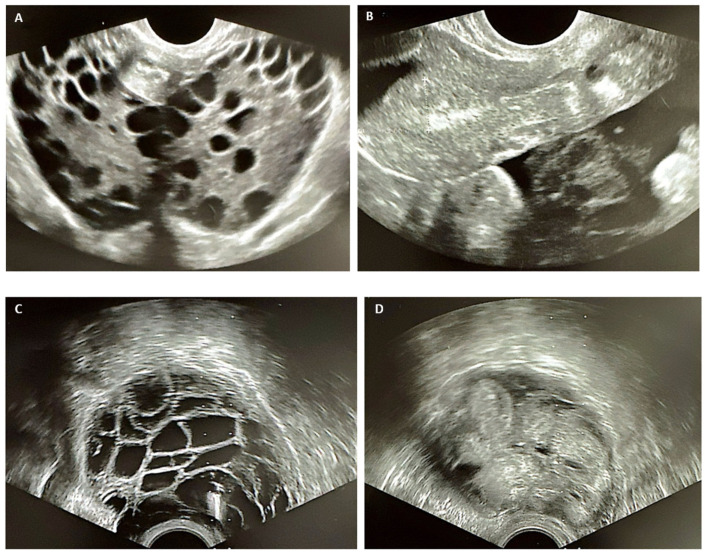
Before starting mitotane therapy for the adrenal cancer, decision of oocyte pick-up and then preservation upon an ovarian stimulation protocol (OSP) was taken, considering the patient’s age, personal option and the fact that post-surgery assessments highlighted no residual disease, nor distant spreading at this point. Fertility preservation plan included OSP followed by oocyte retrieval and oocyte cryopreservation (vitrification technique). Random OSP start was related to the irregular menses profile and the pressure of initiating the adrenolytic agent. OSP included: progestin primed ovarian stimulation (PPOS) with oral progesterone (400 mg/day until trigger day), with long-acting FSH administration (subcutaneous corifollitropin alpha, a single dose of 150 µg in day 1) plus aromatase inhibitor (oral letrozole, 5 mg/day, from day 1 to day 9), followed by final oocyte maturation trigger with gonadotropin releasing hormone (GnRH) agonist (subcutaneous triptoreline, a single dose of 0.2 mg, day 10). Two-month metformin pre-treatment (1000 mg/day) continued during OSP. OSP underwent without complications with a successful pick-up (13 oocytes retrieved and 11 metaphase II mature oocytes were vitrified). Hormonal assays right before oocyte pick-up pointed out: an increase of total testosterone of 6.7 (Normal < 1.67) nmol/L, normal DHEA-S of 224 (Normal: 148–407) µg/dL, FSH of 13.8 mUI/mL, LH of 6.52 mUI/mL, progesterone of 22.4 nmol/L, estradiol of 1184 pg/mL. Glycemia remained normal (< 100 mg/dL) in association with a mildly increased fasting insulin of 23.18 (Normal: 1.9–23) µUI/mL and a HOMA-IR of 4.7. [(**A**,**B**). Pelvic ultrasound scan at the initiation of OSP: right ovary of 3.6 by 2.6 by 2.7 cm, volume of 13.9 mL and left ovary of 4.2 by 2.6 by 2.1 cm, volume of 10 mL, both with polycystic morphology (more than 20 antral follicles); (**B**). Normal uterus with a length of 5.3 cm, antero-posterior diameter of 3.6 cm, and width of 2.7 cm, total volume of 26.9 mL and homogeneous endometrium of 0.63 cm (**C**,**D**). Ultrasound at the end of OSP: (**C**). Utrasound-based ovarian capture at the moment of oocyte pick-up; (**D**). Ultrasound image of the ovary after the ultrasound-guided transvaginal oocyte pick-up procedure]. Figure S7: Flowchart diagram of the timeline perspective in this case. Figure S8. Main hormonal findings according to the real-life setting. In this instance, oncocytic variant of adrenocortical carcinoma represents an exceptional finding among this orphan disease, as it is currently classified in the entire category of adrenal cortex malignancy [1,2,3]. The expected behavior of this particular subtype is heterogenous, due to a very low level of statistical evidence. Some authors suggested a more severe prognosis versus the majority of non-oncocytic (conventional) cases, while others did not confirm a rapidly fulminant outcome [2,3,4,5,6]. However, overall, the tumor embraces an aggressive pattern, as opposite to the oncocytoma, which is a benign tumor of the adrenal cortex, also with > 90% oncocytic cells at pathology exam (which are highly eosinophilic cells with mitochondria-rich cytoplasm). The oncocytic adenoma is situated at the other end of the clinical spectrum, without a risk of recurrence and metastases, the adrenalectomy remaining the single therapy (if any) [1,4]. In oncocytic carcinoma, the current criteria/scores for prognosis and spreading potential are distinct from those of traditional adrenocortical malignancy, according to the 2022 World Health Organization (WHO) classification. The core risk assessment is based on the Lin-WeissBisceglia score [in this case, we confirmed two major criteria (mitoses 6/50 HPF + positive atypical mitoses), while more than one major criterion is consistent with a malignant behavior]. In addition, the analysis of the reticuline algorithm (in this instance: disrupted reticuline network + mitoses 6/50 HPF) correlates with a malignant behavior. Also, the Helsinki score [in this patient: 3 points × 6 (for mitoses 6/50 HPF) + 20 (the value of Ki67) equals 48] also indicated an aggressive outcome, noting that a sum of more than 8.5 is likely corelated with a metastatic potential [4,7,8]. The Weiss score for adrenocortical carcinoma is not adequate in oncocytic variant, nor the extrapolation of insulin growth factor-2 over-expression from traditional adrenocortical carcinomas [1,4,9,10]. Some authors suggested that a level of Ki67 ≥ 20% might serve as an independent poor prognostic factor in this type (while, traditionally, a Ki67 > 5% indicates aggressive adrenal tumor behavior) or the presence of ENSAT (European Network for the Study of Adrenal Tumors) stage III/IV (in this vignette: ENSAT stage II) [11]. Positive beta-catenin staining at immunohistochemistry might help as a supplementary prognostic marker (which was not available in our case) [10]. PHH3 immunostaining may serve as an alternative pointer for mitotic count, especially in adrenal tumors with a low-proliferating profile (in order to distinguish between an oncocytic adenoma and an oncocytic carcinoma) [12].

**Figure 9:**
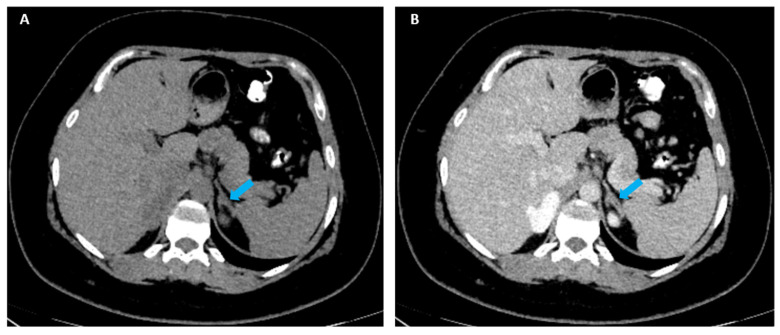
To the best of our knowledge, this is the first case to report an adrenocortical carcinoma in a patient harboring a germline likely pathogenic variant of the *CEL* gene [13,14] in addition with two other somatic pathogenic variants (*NF1+TP53* genes). The true impact of this genetic frame remains an open issue at this point. Generally, the *CEL* gene, located on chromosome 9q34.3, encodes the glycoprotein carboxyl ester lipase (CEL), previously known as cholesterol esterase or bile salt-dependent lipase, an enzyme typically secreted by the acinar cells of the pancreas, responsible for hydrolyzation of lipid esters [15,16,17]. Pathogenic variants of the *CEL* gene cause maturity-onset diabetes of the young type 8 (MODY8), a monogenic, autosomal dominant form (the phenotype includes exocrine pancreatic dysfunction and hereditary pancreatitis) [18,19]. The underlying pathogenic pathways involve abnormal cell cross-talk between pancreatic acinar cells and β-cells, proteotoxicity, dysfunction and loss of β-cell function, as shown by in vitro experiments [20,21] and animal models [22]. Currently, novel *CEL* gene variations are described in relation with early, mild forms of impaired fasting glucose [15,23]. In addition, the gene is potentially expressed in various tissues such as mammary gland, liver cells, blood endothelial cells and macrophages [24], and might show a different expression in some endocrine glands such as ovaries, testes, pituitary and adrenal glands, but, so far, with indeterminate pathological significance [25,26,27]. The present case introduced a likely pathogenic variant, which was most probably associated with an elevated HOMA-IR, while the subject did not develop diabetes until her current age of 20. Moreover, the *NF1* gene, located on chromosome 17q11.2, encodes neurofibromin, a tumor-suppressor protein. Pathogenic germline variants cause neurofibromatosis type 1, a disorder characterized by multiple benign and malignant tumors, including neurofibromas, low-grade glioma, malignant peripheral nerve sheath tumors, breast cancer, pheochromocytoma and gastrointestinal stromal tumors [28,29,30]. While adrenocortical carcinoma is not a typical feature of neurofibromatosis type 1, some authors reported such cases (in germline pathogenic variants) [31]. A recent whole-genome profiling in adrenocortical carcinoma showed key-driver mutations of *APC* and *JAK1* genes, but, also, *TP53* and *NF1*, with no difference in oncocytic versus non-oncocytic variants [32]. Somatic *NF1* mutations have been confirmed in paraganglioma/pheochromocytoma as well [33]. In addition, the *TP53* gene, located on 17p13.1, encodes p53 protein, a tumor suppressor responsible for transcriptional regulation of genes involved in apoptosis, cell-cycle control, DNA repair, metabolism, and immune modulation [34]. Germline pathogenic variants cause Li Fraumeni syndrome, an autosomal dominant cancer susceptibility syndrome characterized by early development of neoplasms including adrenocortical carcinoma, breast cancer, sarcomas and central nervous system tumors [35]. Germline pathogenic variants of *TP53* are also reported in sporadic adrenocortical carcinoma [36], as well as somatic pathogenic variants [37]. Additionally, *NF1*+*TP53* had been reported in one series of NGS-based characterization of adrenocortical malignancy, and positive somatic mutations might serve as prognostic markers in low-grade adrenal cancers, as suggested by some authors [38]. In isolated cases with oncocytic type, somatic variants of the *CTNNB1*, *CDKN2* and *CDKN2B* genes were found [4], or a pathogenic *GNAS R201S* mutation [39], but the current level of evidence remains extremely low. In this instance, another interesting clinical aspect is the overlapping influence on the clinical phenotype, specifically hyperandrogenemia, from ovaries and adrenal glands. Traditionally, as mentioned, the *CEL* gene is related to an abnormal glucose profile amid exocrine pancreas involvement in MODY, which takes place in the absence of insulin resistance and autoimmunity [40]. A limited amount of data suggested *CEL* gene-driven insulin resistance and, in this case, insulin resistance should be regarded as the main factor of polycystic ovary syndrome. *CEL* gene’s role in the development of this adrenal condition has not been reported yet. The mild testosterone elevation during the ovarian stimulation protocol starting from a normal baseline level (as found during post-adrenalectomy evaluation) represents an additional argument for the ovarian source of androgens, and it seems to be a non-cancer-related over-production at this point. Yet polycystic ovary syndrome type A might not be the single cause of hyperandrogenemia in this case, since an elevated level of testosterone and DHEA-s was found a few months before the moment of surgery and immediately normalized after the tumor removal (Figure S8). It is difficult to estimate the actual impact of the adrenal mass as a hormonally active contributor to the entire clinical picture during the patient’s prior teenager years. Similarly, the natural history of the tumor growth and development has not been registered due to a lack of serial imaging evaluation in the meantime. Moreover, acne, obesity and irregular menses might mimic Cushing’s syndrome as well. This case history belonged to a real-life setting, and we retrospectively found no particular investigations over the years, nor did we had pre-operatory dexamethasone suppression testing in order to assess the endogenous hypercortisolemia. However, the absence of post-operatory adrenal insufficiency was consistent with glucocorticoid axis non-involvement. Additionally, a late-onset congenital adrenal hyperplasia should be ruled out. Post-adrenalectomy, the left adrenal gland presented a mild hyperplasia, as shown on the CT scan. [(**A**,**B**). Post-operatory CT scan: (**A**). native phase, axial plane: left adrenal gland (blue arrow) with size at the upper normal limit, a convex shape, and mildly lobulated margins, suggesting an adrenal hyperplasia; (**B**). delayed phase, axial plane]. Yet normal 17-hydroxyprogesterone has been found by the age of 19. *CYP* gene screening was also negative and post-adrenalectomy we performed short Synacthen (tetracosactide) testing, revealing a non-significant raise of 17-hydroxyprogesterone, from a baseline value of 0.85 ng/mL to a maximum of 1.46 ng/mL (of note, the testing interpretation in individuals with a single adrenal gland remains out of the current standards). Other than hyperandrogenemia, isolated reports of oncocytic adrenal cancers presented Cushing’s syndrome [39], resistant endocrine hypertension with hypokalemia [41], ectopic parathyroid hormone production and estradiol excess [42]. Distinct (abnormal) pathways to associate glucose/insulin profile anomalies had not been reported. We mentioned a case of hyperandrogenemia associated with the absence of glucose transporter type 1 (GLUT1) in an adrenal malignant tumor, which generated false negative results at ^18^F-fluorodeoxyglucose uptake at PET [43]. To conclude, this case highlights a novel genetic frame (germline likely pathogenic variant of *CEL* gene and somatic pathogenic variants of *NF1*+*TP53* genes) in the challenging field of adrenal cancer, which requires a multidisciplinary awareness from an early age [44]. The exceptional oncocytic variant overlapped with polycystic ovary syndrome type A, and both might be involved in the clinically and biochemically manifested hyperandrogenemia. Whether thymus enlargement is part of this gene-related landscape remains an open matter, but so far it seems an incidental finding without clinical significance. Yet previous rare reports identified thymic hyperplasia/tumors after adrenal tumor removal and/or correction of the associated hormonal excess, an aspect that is yet to be understood [45,46,47]. Notably, due to the rarity of this distinct endocrine entity, the overall management can only be tailored to the case in point, while taking into consideration the general approach for malignancies of the adrenal cortex (surgery followed by mitotane and even platinum-based chemotherapy in advanced stages) [5]. Current ENSAT guidelines are pivoting surgery, if feasible (which should only be performed by highly experienced surgeons in this specific field), followed by adjuvant mitotane therapy after radical resection, upon a multidisciplinary workup. In case of a local recurrence (after at least one year), re-do surgery should be taken into account, as well as surgery for single metastatic disease, while a rapid progression, multi-metastatic disease of incomplete surgical removal, recommends chemotherapy or even immune therapy. In this young patient, only mitotane was recommended based on the post-operatory imaging evaluation that showed complete tumor resection, no disease spreading, nor early post-adrenalectomy recurrence [5,48,49,50]. Hence, to our best knowledge, this is the first case of oncocytic adrenocortical carcinoma undergoing an oocyte pick-up and preservation procedure before starting the adrenolytic medication (noting the general good health status and the lack of a residual disease at this point), which was meant to assist further reproductive potential in case of an overall good prognosis and depending on patient’s options.

## Data Availability

The original contributions presented in this study are included in the article/Supplementary Materials. Further inquiries can be directed to the corresponding authors.

## Supplementary Materials

The following supporting information can be downloaded at https://www.mdpi.com/article/10.3390/diagnostics16121935/s1, Figure S1: supplementary data for Figure 2C: blue arrow = pleomorphic nuclei; orange arrow = atypical mitosis title; Figure S2: Supplementary data for Figure 2D: green cercle = atypical mitosis; Figure S3: Supplementary data for Figure 3C: red arrow = nuclear positive reaction for SF1; Figure S4: Supplementary data for Figure 3D: yellow arrow = positive reaction in the tumor cells for Ki67; Figure S5. Integrative Genomics Viewer (IGV version 2.16.0) for the *CEL* gen; Figure S6. c.539-2A>G; Figure S7. Flowchart diagram of the timeline perspective in this case; Figure S8. Main hormonal findings according to a real-life setting.

## Abbreviations

The following abbreviations are used in this manuscript:

ACTH	adrenocorticotropic hormone
ACMG	American College of Medical Genetics and Genomics
CT	computed tomography
CEL	carboxyl ester lipase
DHEA-S	dehydroepiandrosterone sulphate
ENSAT	European Network for the Study of Adrenal Tumors
FSH	follicle-stimulant hormone
GnRH	gonadotropin releasing hormone
GLUT1	glucose transporter type 1
HPF	high power field
HOMA-IR	Homeostatic Model Assessment of Insulin Resistance
LH	luteinizing hormone
MRI	magnetic resonance imaging
MMR	Mismatch Repair Proteins
MSS	microsatellite stability
MSI	microsatellite instability
MODY	maturity-onset diabetes of the young
NGS	next-generation sequencing
PET/CT	positron emission tomography/computed tomography
PPOS	progestin primed ovarian stimulation
PHH3	phospho-histone H3
OSP	ovarian stimulation protocol
OGTT	oral glucose tolerance test
SF1	steroidogenic factor 1
WHO	World Health Organization
