# Supplementary material for: Oncocytic Adrenocortical Carcinoma with Somatic Pathogenic Variants of NF1 and TP53 Genes in a Young Adult Harboring a Germline Likely Pathogenic Variant in CEL Gene: From Hyperandrogenemia of Dual (Adrenal–Ovarian) Cause to Oocyte Preservation and Mitotane Initiation
Source: Diagnostics (Basel). 2026 Jun 22;16(12):1935. doi: 10.3390/diagnostics16121935 (PMC13297793; doi:10.3390/diagnostics16121935)

Supplementary Materials:

Figure S1: Supplementary data for Figure 2C: blue arrow = pleomorphic nuclei; orange arrow = atypical mitosis

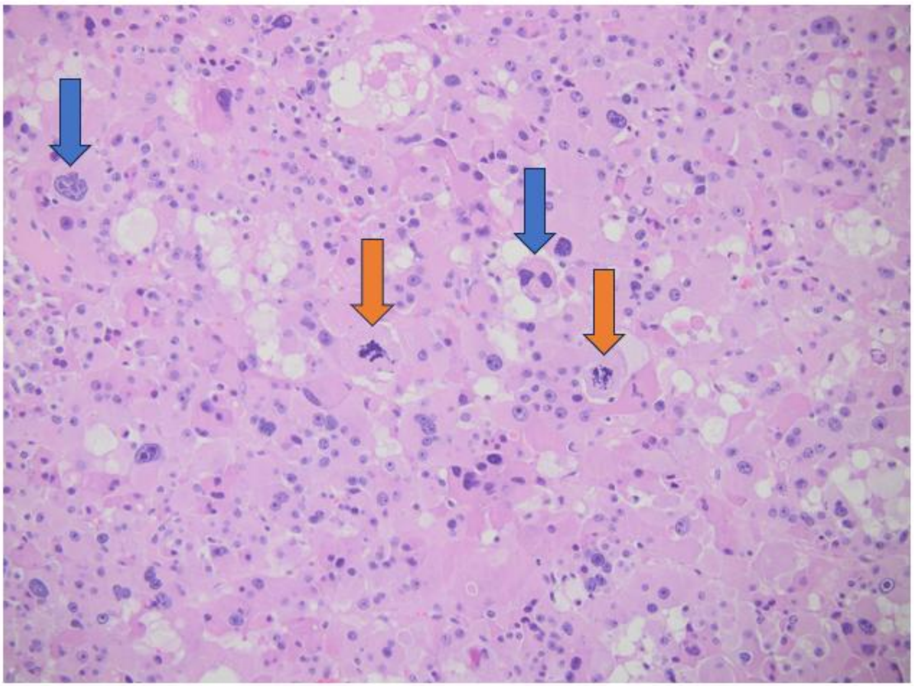

Figure S2: Supplementary data for Figure 2D: green cercle = atypical mitosis

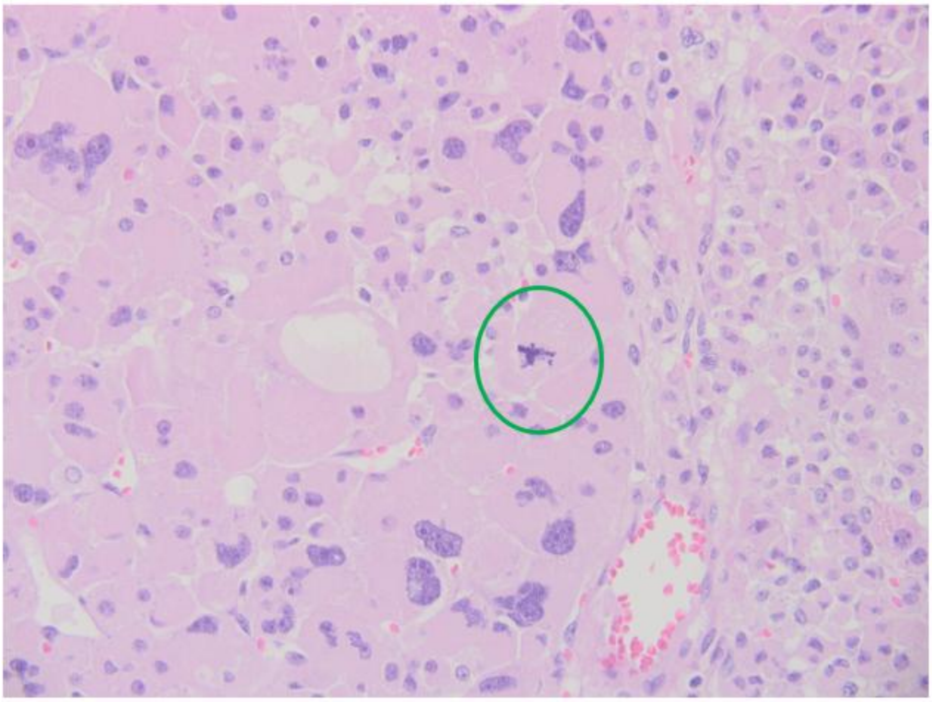

Figure S3: Supplementary data for Figure 3C: red arrow = nuclear positive reaction for SF1

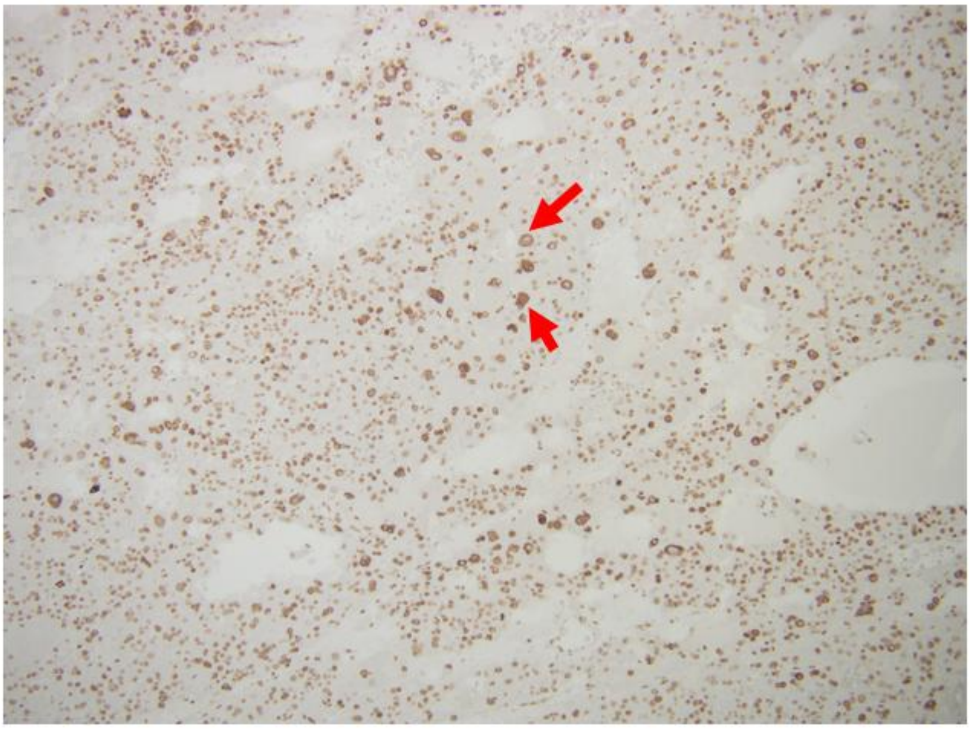

Figure S4: Supplementary data for Figure 3D: yellow arrow = positive reaction in the tumor cells for Ki67

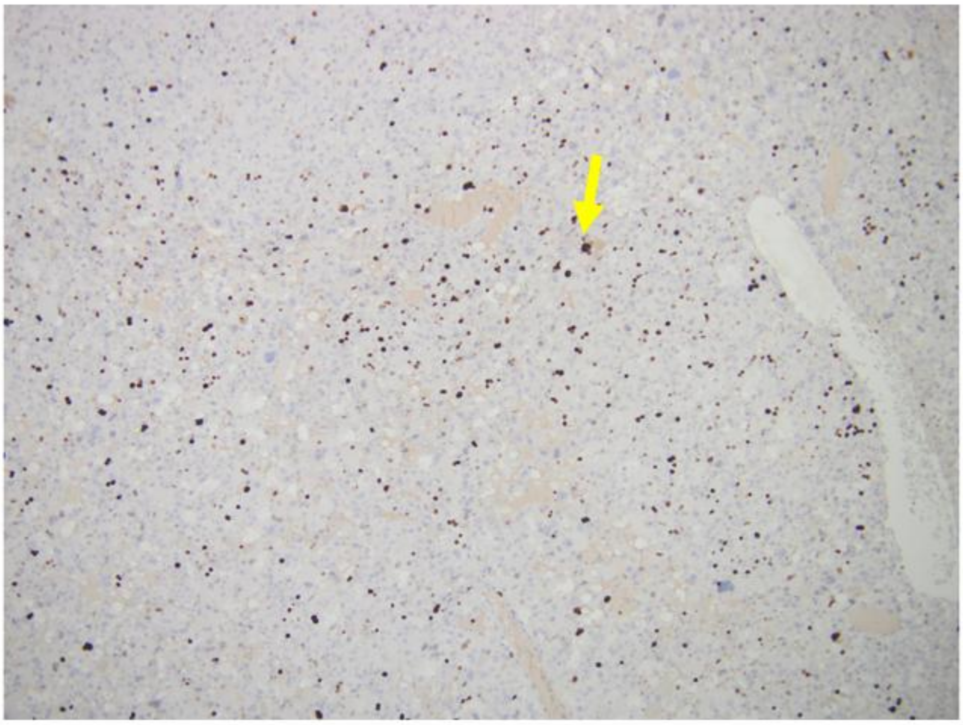

Figure S5. Integrative Genomics Viewer (IGV version 2.16.0) for the *CEL* gen

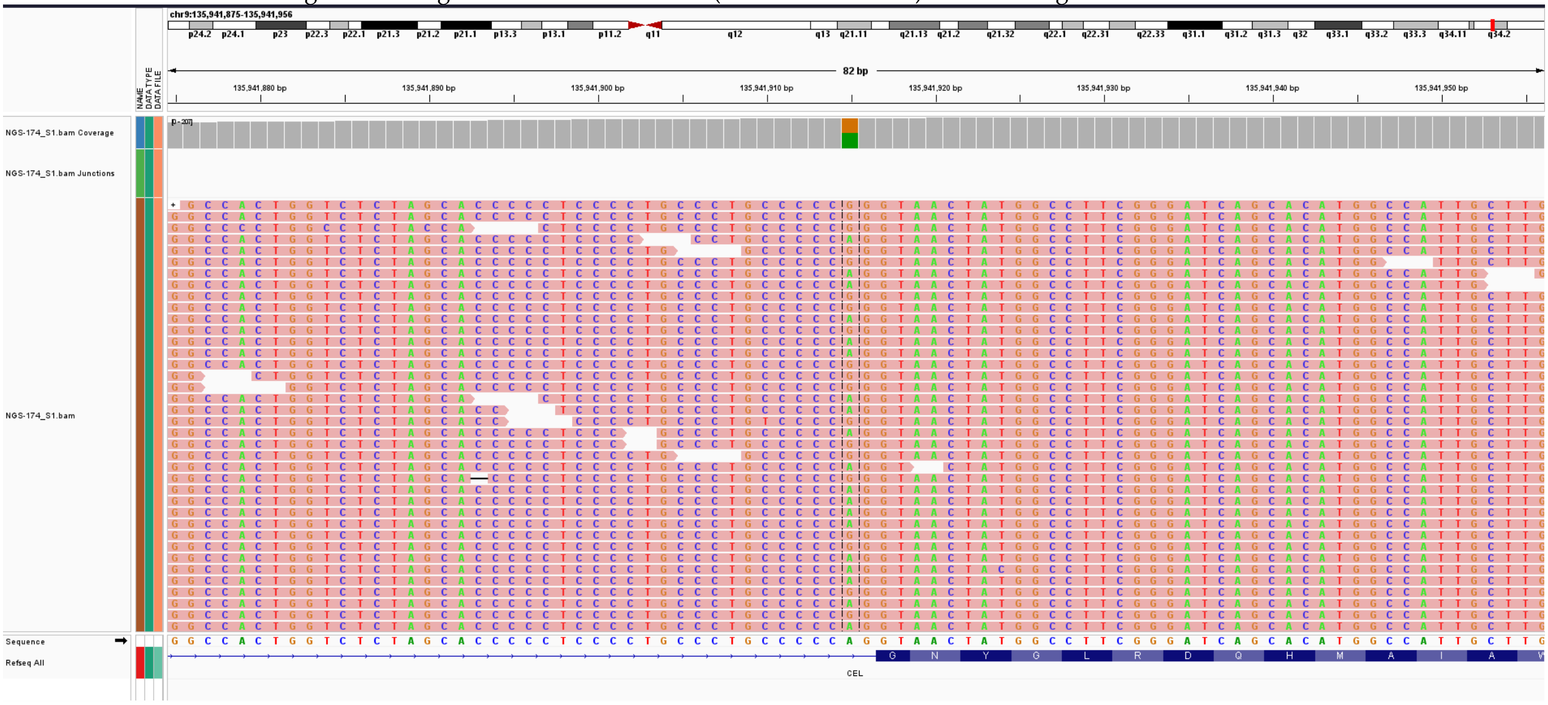

Figure S6. c.539-2A>G

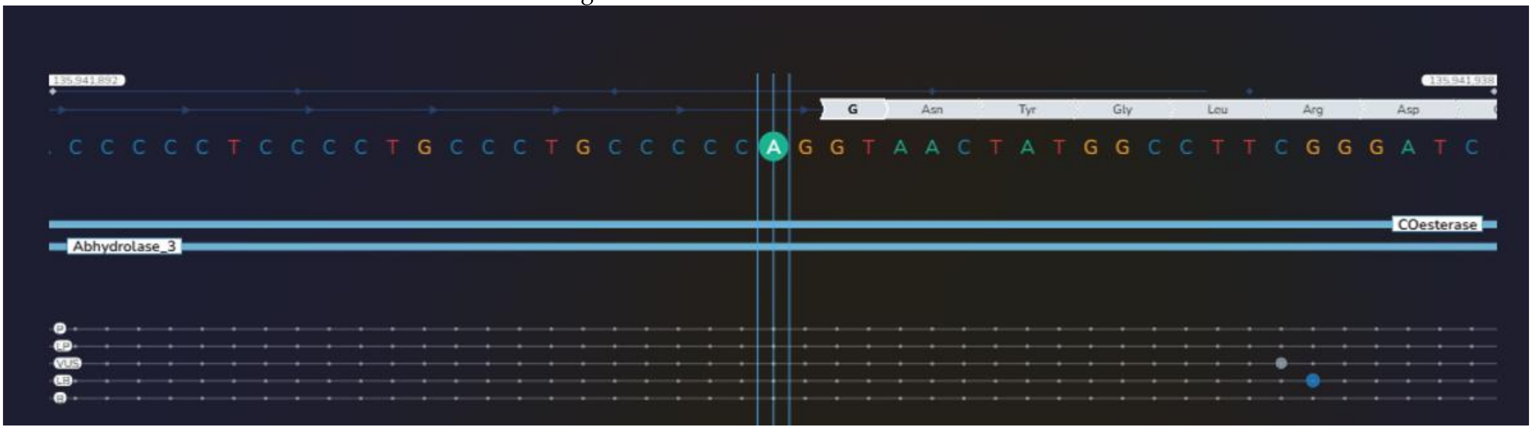

Figure S7. Flowchart diagram of the timeline perspective in this case

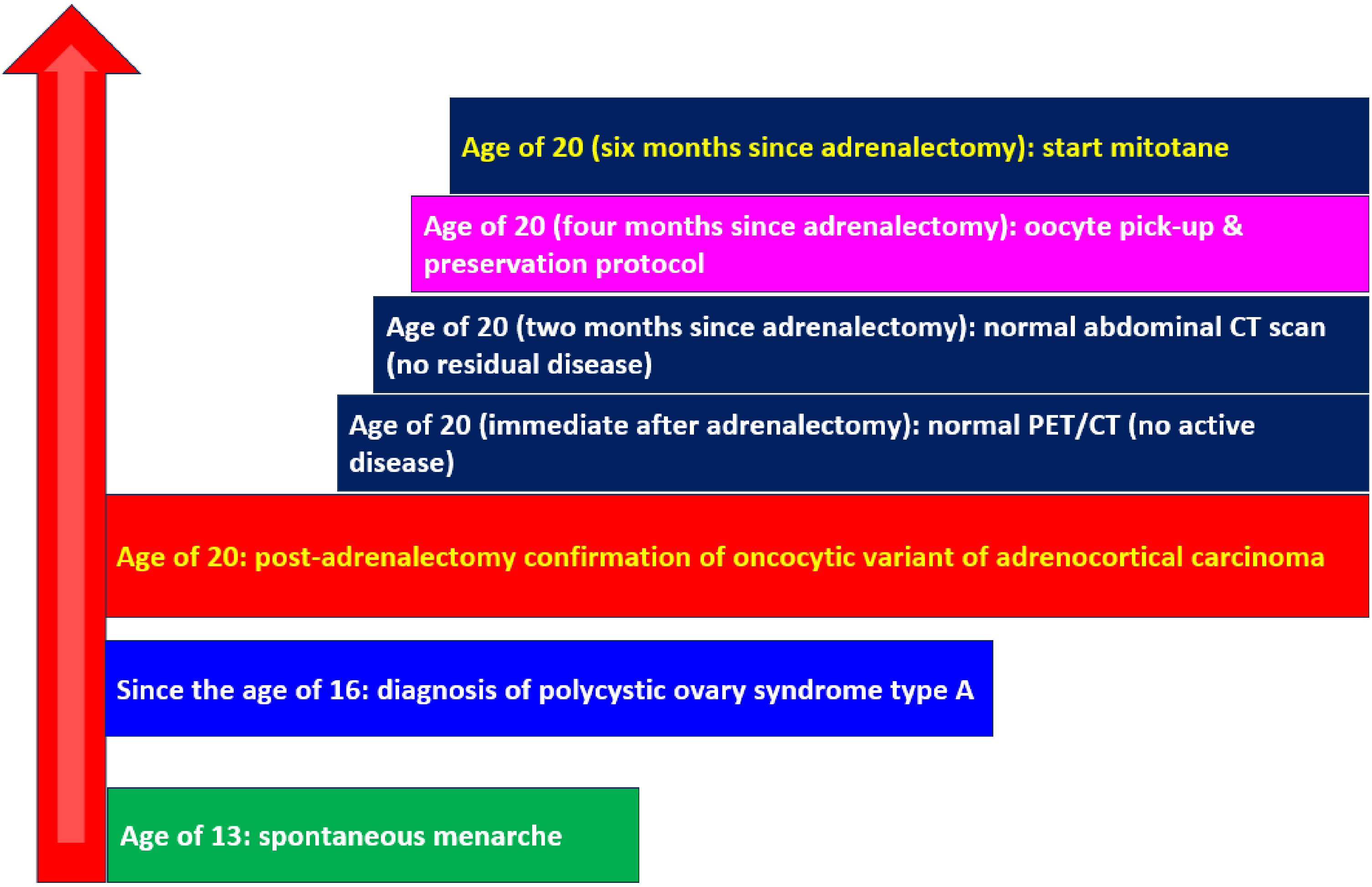

Figure S8. Main hormonal findings according to a real-life setting

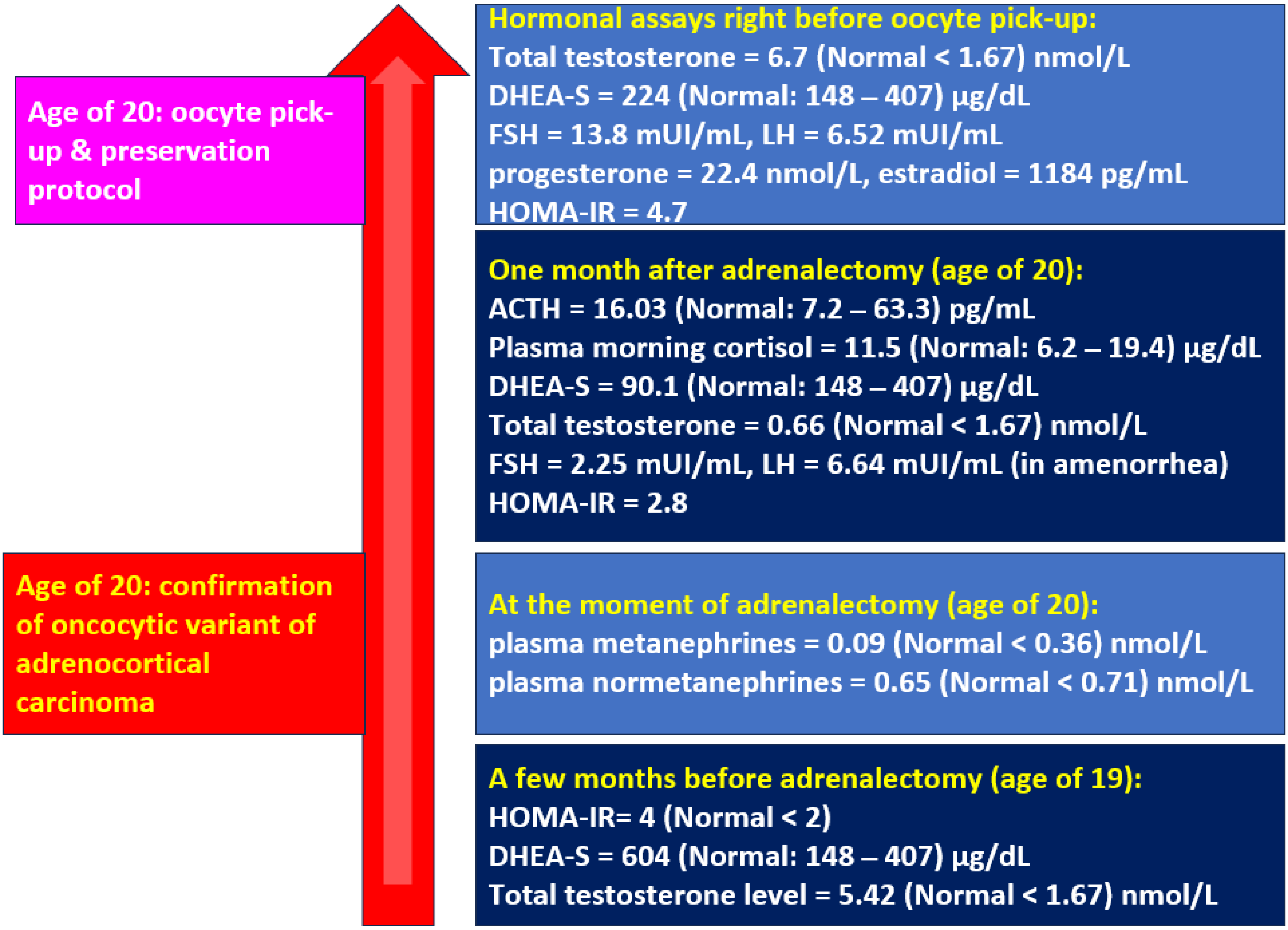

Supplement: Supplementary file 1 [file diagnostics-16-01935-s001.zip › diagnostics-4273182-supplementary.pdf]
